# Which patient to operate? The ethics of providing heart surgery in developing countries

**DOI:** 10.1186/1749-8090-8-S1-O254

**Published:** 2013-09-11

**Authors:** K Fenton

**Affiliations:** 1 International Children’s Heart Foundation, Memphis, TN, USA

## Background

Approximately 90 percent of the world’s population lives in countries with little or no access to cardiac surgery. Initiation of a surgical program results in a large influx of patients, of whom only a small proportion can be selected for surgical intervention. In addition, at times program development goals may conflict with patient care goals. When surgery cannot be provided to all the patients who need it, it is worthwhile to examine the ethics of patient selection.

## Methods

The difficulties regarding patient selection are analyzed according to the two most commonly used ethical systems (utilitarian ethics and virtue ethics).

## Results

In utilitarian ethics, patients are chosen in order to optimize the “good” for the entire population. Selection according to this system would aim not so much to operate on the largest number of patients at the lowest per-patient cost (a common misconception), but rather to use the resources available to relieve the most suffering and improve longevity. Genuine application of utilitarian ethics awaits accurate analysis of the cost effectiveness of heart surgery in individual settings. In its purest form, utilitarian ethics would promote program development over concern for individual patients, even to the point of “sacrificing” patient good for the benefit of the program, since this would benefit the largest number in the long term. By stark contrast, virtue ethics seeks to provide the best possible solution for each individual patient. Paramount importance is given to the good of person; the good of the group as a whole only enters by way of determining what resources are available. An individual patient can never be “used” or “sacrificed” to benefit the group.

## Conclusions

In cardiothoracic surgery, it is the surgeon’s responsibility to provide the best possible care for each one of his patients. The ethical system best used as a guide to patient “selection” in situations of limited resources is virtue ethics.

